# Therapeutic Collaboration in Career Construction Counseling: Case Studies of an Integrative Model

**DOI:** 10.3389/fpsyg.2021.784854

**Published:** 2022-02-02

**Authors:** Filipa Silva, Maria do Céu Taveira, Paulo Cardoso, Eugénia Ribeiro, Mark L. Savickas

**Affiliations:** ^1^School of Psychology, University of Minho, Braga, Portugal; ^2^Department of Psychology, University of Évora, Évora, Portugal; ^3^Department of Family and Community Medicine, Northeast Ohio Medical University, Rootstown, OH, United States

**Keywords:** career counseling, career construction, case study, coding system, change process, career narrative, good outcome, therapeutic collaboration

## Abstract

The mapping of therapeutic collaboration throughout counseling deepens our understanding of how the helping relationship fosters client change. To better understand the process of career construction counseling (CCC), we analyzed the therapeutic collaboration on six successful face-to-face cases. The participants were six Portuguese adults, five women and one man, real clients of a career counseling service, and four psychologists, three female and one male trained in the career intervention model. The participants completed demographic questions and measures of career certainty, vocational identity, career indecision, and psychological functioning. The Therapeutic Collaboration Coding System was used to track collaboration throughout all interactive episodes. The clinical significance of the intervention was calculated by analyzing pre-post-test statistical differences for each case, with the Reliable Change Index and *Z* score. The findings evidenced a pattern of therapeutic collaboration evolution for good outcome cases. Based on this pattern, we propose a model of process-outcome evolution for the three phases of CCC.

## Introduction

Career construction counseling (CCC; Savickas, 2019a) is a narrative practice that emphasizes the relational and narrative processes of career construction. In the CCC model, counselors are encouraged to foster the resolution of clients’ career concerns, by collaborating and supporting clients as they rewrite a continuous and coherent life narrative. The intervention occurs throughout three phases. In the first phase, counselors facilitate problem formulation and conduct a career construction interview (CCI; Savickas, 2015) to evoke life story episodes. In this phase, counselor and client also begin the exploration of career constructs, such as needs, interests, goals and adaptability resources. The CCI consists of five questions about (1) role models, to help the client evoke self-narrative in the future, (2) favorite TV programs, magazines or internet sites, to identify preferred occupational settings, (3) current favorite story, to hear from clients the description of core life problems and how they deal with them, (4) mottos, to evoke the advice clients give to themselves when facing life/career challenges, and (5) early recollections, to unfold the clients’ view of the current career concern (Cardoso et al., 2019; Savickas, 2019a). During the second phase, the counselor and client explore the meanings contained in the responses to CCI, to reauthor the career narrative as a basis for new career plans. In the third phase, clients evaluate the realism of career plans, consider how to put their intentions into action, and reflect upon the changes achieved during the intervention (Cardoso et al., 2019; Savickas, 2019a; Silva, 2020).

The outcome research has shown the effectiveness of CCC in fostering career adaptability (Barclay and Stoltz, 2016; Ginevra et al., 2017; Santilli et al., 2019; Souza and Teixeira, 2020), resolving career decision-making difficulties (Di Fabio and Maree, 2012; Obi, 2015; Barclay and Stoltz, 2016; Cardoso et al., 2016, 2017), increasing resilience (Santilli et al., 2019), promoting reflexivity (Souza and Teixeira, 2020), and promoting feelings of well-being and confidence (Obi, 2015). Process research based in the innovative moments’ perspective of clients’ narrative change (IMM; Gonçalves et al., 2009) indicated that, throughout CCC, the clients’ narrative transformation evolves from the understanding of career problems to the elaboration of a life story macro-narrative. Then, clients’ narrative change moves toward both the construction of new career plans and the understanding of changes achieved during the intervention (Cardoso et al., 2014a,b, 2016).

Despite the importance and focus on the collaborative relationship for meaning making in CCC, research on this process dimension is scarce (e.g., Cardoso et al., 2016; Taveira et al., 2017; Silva, 2020). Responding to this gap, the present study analyzed how the therapeutic collaboration between counselor and clients evolves during the three phases of CCC. This knowledge is fundamental to understand how therapeutic collaboration fosters clients change. In conducting this research, we used the conceptual and methodological tools offered by the Therapeutic Collaboration Model (TCM; Ribeiro et al., 2013).

The TCM is rooted in psychotherapy research. It emphasizes that therapeutic collaboration evolves during the process of interaction between counselor and client across each counseling session. The analysis of therapeutic collaboration occurs at a micro-analytical level, addressing, moment-by-moment, the coordination of actions between counselor and client, toward the client’s change (Ribeiro et al., 2013).

**FIGURE 1:**
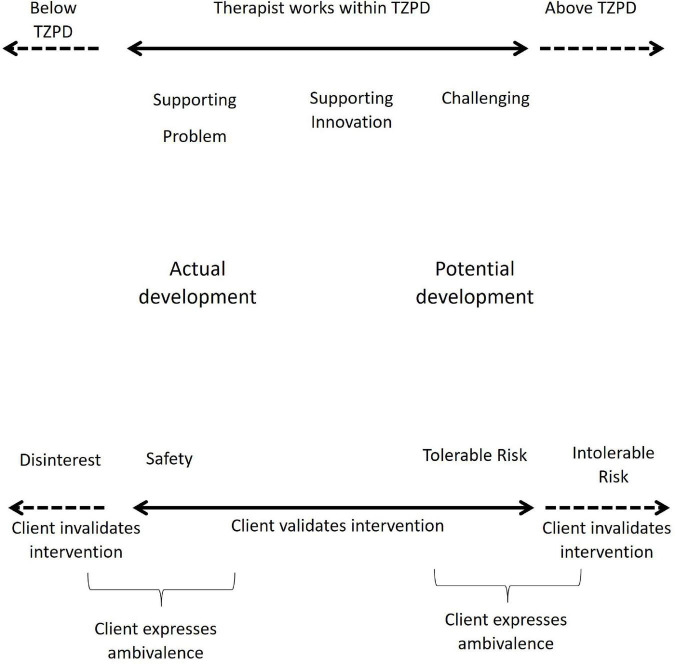
Therapeutic collaboration model. From: *How collaboration becomes therapeutic: The therapeutic collaboration coding system*, by Ribeiro et al. (2013). Adapted with permission.

Drawing on the concepts of validation (Kelly, 1955) and proximal development zone (Vygotsky, 1978), Ribeiro et al. (2019) suggests that the therapeutic collaboration can be viewed as a mutual validation process along a developmental track of assisted change. According to this view, counselor and client are expected to use empathetic comprehension of mutual experiences in the counseling process. At the same time, they are supposed to achieve mutual understanding of the client’s actual difficulties, the recognition and amplification of the client’s resources and potential for change, as well as the co-construction of new meanings and possible opportunities for the client’s change. Accordingly, the TCM establishes that change occurs when counselors challenge clients to take a different perspective on the initial problematic self-narrative and make a new meaning about one’s own career story. In doing so, clients move toward their therapeutic zone of potential development (TZPD; Ribeiro et al., 2013, 2019). The TCM is presented in more detail in Figure 1.

From this perspective, the dyad works collaboratively into the client’s TZPD, when the client validates the counselor interventions, either because these interventions support the client’s initial problematic narrative (a safety impact), or because the client feels potentially ready to change, supported by the counselors (tolerable risk impact). When the client invalidates the counselor’s intervention, they work non-collaboratively, outside the client’s TZPD. The clients may also respond with ambivalence when they simultaneously validate and invalidate the counselor in the same action.

The TCM informed the Therapeutic Collaboration Coding System (TCCS; Ribeiro et al., 2013), developed to intensively assess therapeutic collaboration. This coding system is used to consider each individual unit of analysis, that is, every speaking-turn in a counselor’s intervention and the client’s response. Tables 1, 2 display the categories and subcategories of the counselor and client’s interventions. Every unit of analysis is coded with the categories and sub-categories presented in Tables 1, 2. After coding every speaking-turn, each intervention is coded in relation to the TCM (Ribeiro et al., 2013). Counselors’ interventions are coded as Support Problem, Support Innovation and Challenge. Counselors support clients when they reflect upon the content of clients’ speech. The way counselors support clients is different concerning the clients’ developmental level in the TZPD (Ribeiro et al., 2013) and the content of clients’ narratives. When counselors reflect upon clients’ problematic self-narratives, the interventions are coded as Supporting Problem. When counselors reflect upon a new meaning that emerged in clients’ narratives, the interventions are coded as Supporting Innovation. Concerning the TZPD (Ribeiro et al., 2013) when counselors support the problem, clients are at their actual development level and when counselors support innovation, clients are moving to their potential development level. Counselors challenge clients when they extend the meaning of the content of clients’ speech and invite clients to take a different perspective on their problems. Challenge interventions invite clients to respond at their potential development level.

**TABLE 1:** Therapeutic collaboration coding system: therapist intervention coding categories and sub-categories.

Supporting	Definitions
Reflecting	The therapist reflects the content; meaning or feeling present in the client’s words. He or she uses his/her or client’s words but does not add any new content in the reflection, asking for an implicit or explicit feedback.
Confirming	The therapist makes sure he/she understood the content of the client’s speech, asking the client in an explicit and direct mode.
Summarizing	The therapist synthesizes the client’s discourse, using his/her own and client’s words, asking for feedback (implicit or explicit).
Demonstrating interest/attention	The therapist shows/affirms interest on client’s discourse.
Open questioning	The therapist explores clients’ experience using open questioning. The question opens to a variety of answers, not anticipated and/or linked to contents that the client does not reported or only reported briefly. This includes the therapist asking for feedback of the session or of the therapeutic task.
Minimal encouragement	The therapist makes minimal encouragement of the client’s speech, repeating client’s words, in an affirmative or interrogative mode (ambiguous expressions with different possible meanings are not codified, like a simple “Hum. hum” or “ok”).
Specifying information	The therapist asks for concretization or clarification of the (imprecise) information given by the client, using closed questions, specific focused questions, asking for examples.

**Challenging**	**Definitions**

Interpreting	The therapist proposes to the client a new perspective over his or her perspective, by using his or her own words (instead of the client’s words). There is, however, a sense of continuity in relation to the client’s previous speaking turn.
Confronting	The therapist proposes to the client a new perspective over his or her perspective, or questions the client about a new perspective over his or her perspective. There is a clear discontinuity with (i.e., opposition), in relation to the client’s speaking turn.
Inviting to adopt a new perspective	The therapist invites (implicitly or explicitly) the client to understand a given experience, as an alternative.
Inviting to put into practice a new action	The therapist invites the client to act in a different way, in the session or out of the session.
Inviting to explore hypothetical scenarios	The therapist invites the client to imagine hypothetical scenarios, that is, cognitive, emotional, and/or behavioral possibilities that are different from the client’s usual way of understanding and experiencing.
Changing level of analysis	The therapist changes the level of the analysis of the client’s experience, from the descriptive and concrete level to a more abstract one or vice versa.
Emphasizing novelty	The therapist invites the client to elaborate upon the emergence of novelty.
Debating client’s beliefs	The therapist debates the evidence or logic of the client’s beliefs and thoughts.
Tracking change evidence	The therapist searches for markers of change and tries to highlight them.

*From: How collaboration becomes therapeutic: The therapeutic collaboration coding system, by E. Ribeiro et al. (2013). Adapted with permission.*

**TABLE 2:** Therapeutic collaboration coding system: client’s response coding categories and sub-categories.

Validation	Definitions
Confirming	The client agrees with the therapist’s intervention, but does not extend it.
Giving information	The client provides information according to therapist’s specific request.
Extending	The client not only agrees with the therapist intervention, but also expands it (i.e., going further).
Reformulating oneself perspective	The client answers the therapist’s question or reflects upon the therapist’s prior affirmation and, in doing so, reformulates his or her perspective over the experience being explored.
Clarifying	The client attempts to clarify the sense of his or her response to the therapist’s prior intervention or clarify the sense of the therapist’s intervention itself.

**Invalidation**	**Definition**

Expressing confusion	The client feels confused and/or states his or her incapacity to answer the therapist’s question.
Focusing/persisting on the dominant maladaptive self-narrative	The client persists on looking at a specific experience or topic from his or her standpoint.
Defending oneself perspective and/or disagreeing with therapist’s intervention	The client defends his/her thoughts, feelings or behavior, by using self-enhancing strategies or self-justifying statements.
Denying progress	The client states the absence of change (novelty) or progress.
Self-criticism and/or hopelessness	The client is self-critical or self-blaming and becomes absorbed in a process of hopelessness (e.g., client doubts about the progress that can be made).
Lack of involvement in response	The client gives minimal responses to the therapist’s efforts to explore and understand the client’s experience.
Shifting topic	The client changes topic or tangentially answers the therapist.
Topic/focus disconnection	The client persists in elaborating upon a given topic, despite the therapist’s efforts to engage in the discussion of a new one.
Non-meaningful storytelling and/or focusing on others’ reactions	The client talks in a wordy manner or overly elaborates non-significant stories to explain an experience and/or spends an inordinate amount of time talking about other people.
Sarcastic answer	The client questions the therapist’s intervention or is ironic toward the therapist’s intervention.

*From: How collaboration becomes therapeutic: The therapeutic collaboration coding system, by Ribeiro et al. (2013). Adapted with permission.*

Clients’ responses are coded as disinterest, safety, tolerable risk, ambivalence and intolerable risk. Disinterest and Intolerable Risk responses correspond to invalidation responses. The difference between them refers to the development level of the clients at that time and the way clients respond to counselors. Safety and Tolerable Risk responses correspond to validation responses. Safety responses include confirming and giving information and refer to clients’ validation in their current development level. Tolerable Risk responses include extending and reformulating oneself perspective and refer to clients’ validation in their potential development level, when clients move forward toward change. Ambivalence is a response that includes a validation and an invalidation at the same time, in the same intervention.

The crossover of the counselor’s interventions and the client’s responses result in 15 different types of therapeutic interactions. These interactions can be collaborative or non-collaborative in reference to the client’s TZPD.

The TCCS is designed to be applied to each interaction across an entire session, and then to the entire counseling intervention.

The TCCS reliability was obtained by Ribeiro et al. (2013) through the analysis of the intervention process of five clients in psychotherapy, with a total of 82 therapeutic sessions. The internal consistency of the study was 0.92 for counselors’ interventions and 0.93 for clients’ responses (Ribeiro et al., 2013). The TCCS reliability has consistently shown good results with different therapeutic approaches and outcomes: narrative therapy (Ribeiro et al., 2014; Ferreira et al., 2015; Pinto et al., 2018); constructivist therapy (Ribeiro et al., 2018); cognitive-behavioral therapy (Pires, 2016; Stiles et al., 2016; Rodrigues, 2018); emotion focused therapy (Ribeiro et al., 2016a); client centered therapy (Ribeiro et al., 2014); good and poor outcome and dropout cases (Ribeiro et al., 2014, 2016a; Ferreira et al., 2015; Pires, 2016; Stiles et al., 2016; Pinto et al., 2018; Rodrigues, 2018).

Taveira et al. (2017) were also successful in applying the TCCS for the first time to a single case of CCC.

The present study investigated the following research question: From the TCM, how does therapeutic collaboration evolve throughout CCC intervention?

We used a holistic multiple case study design since each case was the main unit of analysis (Yin, 2009). Considering the exploratory nature of this study, cases were selected to have a wide picture on the evolution of collaboration during CCC. Hence, we organized two groups. One with three participants who revealed statistically significant change in all measures of vocational behavior. The second group, also with three participants, revealed modest changes in vocational behavior (statistically significant change in two, one and none measure of vocational behavior, respectively).

By intensively analyzing therapeutic collaboration in six successful face-to-face CCC cases, this study adds to career counseling theory, research and practice. The identification of a therapeutic collaboration sequence during CCC contributes to deepen our understanding on the role of counselor/client relationship to foster meaning making, here defined as a process by which clients “structure and give meaning to uncertain or ambiguous situations” (Savickas, 2019b, p. 10). This knowledge is relevant to guide counselors’ practice in the management of relational and technical dimensions of their interaction with clients. Finally, using the TCCS, this study extends its applicability to career counseling, opening new research possibilities in this field.

## Materials and Methods

### Participants

#### Clients

The participants were six Portuguese adults, five women and one man, real clients of a career counseling service, in two public universities and a private clinic. The sociodemographic characteristics of the six clients are presented in Table 3. Two of the participants were 18 years old and the others were 22, 23, 28, and 41 years old. One client completed 11 years of schooling, two clients completed higher education, two were graduates and one completed a master degree. During the period of the study, prior to the pandemic, five participants were students and one was unemployed. The participants were selected from a pool of 43 participants in a larger study on CCC process and outcome. The selection criterion was the pattern of outcomes obtained with the intervention from cases with pre–post test measures (dropout cases were not included) and videotaped in good conditions. The selected cases for in-depth analysis of the CCC process were representative of the different outcome patterns obtained in the larger sample. Two groups were organized: one with three participants who revealed statistically significant change in all measures of vocational behavior. The second group, also with three participants, revealed modest changes in vocational behavior (statistically significant change in two, one and none measure of vocational behavior, respectively).

**TABLE 3:** Sociodemographic characteristics of the six clients.

Client	Gender	Age	Degree	Employment status
Cecília	Female	28	Master Degree	Student
Nuno	Male	23	Graduation	Unemployed
Diana	Female	18	Higher education	Student
Olga	Female	22	Graduation	Student
Vanda	Female	41	Higher education	Unemployed
Tatiana	Female	18	11th grade	Student

Cecília is a 28 years-old woman, Ph.D. student that seek counseling to have support in her next career decision concerned with the end of her Ph.D. Cecília is a kindergarten teacher, and she is struggling with the choice between a different area like kindergarten management, where it could be difficult to get a job or continue to be a kindergarten teacher accepting the career stagnation that choice could bring.

Olga is a 22-years-old woman, MsD student that looked for counseling because of her uncertainty about the area of her degree she would like to work in the future and in which she could feel more useful. Olga feels she lacks practical experience, and she is insecure about her skills for being a good professional in the future.

Vanda is a 41-years-old unemployed woman who seek counseling because of her unemployment situation. She used to work in the commercial area, but she feels tired, and she wants to understand the reasons why she remains unemployed. She recognizes that change will be complex and difficult due to her age (42) and her lack of experience in any other area.

Nuno is a 23-year-old man that seek counseling because of the career transition he was facing at the moment. He ended his degree and is in a decision process between entering the labor market or entering a MsD. Nuno is very confused and doesn’t want to work in his degree. Nuno sees in counseling an opportunity to stop and think about his life but is skeptical about career counseling efficacy.

Diana is an 18-years-old female student that seek counseling to get a better knowledge about herself and to look for a different area of study, once she considered she didn’t search many options and she didn’t make a well-informed decision. Diana sought professional help to make a better-informed decision.

Tatiana is an 18-years-old student that looked for counseling because she entered university without her family consent and that went wrong. She looked for professional help to make a better career decision, more informed and thoughtful.

Since this is an exploratory phase of research on the role of therapeutic collaboration for meaning making, one of our intentions was to have different types of good outcome cases to identify communalities in the evolution of therapeutic change.

#### Counselors

Four psychologists (three female and one male) conducted the intervention. The female psychologists were 26, 31, and 48 years old, the first two with a master’s degree and the last with an undergraduate degree, with 4, 8, and 17 years of professional experience, respectively. The male psychologist was 54 years old, with a Ph.D. and with over 20 years of professional experience.

#### Researchers and Training

Two judges coded the intervention sessions and an auditor was available to code in case of disagreement by the judges. One judge was the first author of this article and the other judge was a 30-year-old Caucasian Ph.D. student in Psychology, co-author of the TCCS manual (Ribeiro et al., 2016b). The auditor was a female psychologist with a Ph.D. in Clinical Psychology, and the main author of the Therapeutic Collaboration Model and its system (TCCS; Ribeiro et al., 2013). The judges’ training involved: (1) Reading the relevant literature on TCM (Ribeiro et al., 2013) and TCCS (Ribeiro et al., 2013), (2) independent coding and reaching of agreement in four sessions of two clinical cases of psychotherapy. The training was completed when agreement percentage reached 80%, both for the therapists’ interventions and clients’ responses. The judges’ training was organized and guided by the author of the TCCS (Ribeiro et al., 2013).

### Measures

*Demographic information* was collected using a brief questionnaire in which participants reported their age, gender, education, occupational condition and main career concern.

*Career Certainty* was assessed with the *Vocational Certainty Scale* (VCS; Santos, 2007), with four items (e.g., “I have already made my career choice and do not intend to change”) responded on a 6-point scale ranging from (1) strongly disagree to (6) strongly agree. Alpha coefficients in a Portuguese students’ sample is reported as 0.85 (Santos, 2007). The VCS has been shown to positively correlate with vocational identity and self-esteem, and negatively with indecision and anxiety (Santos, 2007).

*Vocational identity* was measured using the *Vocational Identity Scale* (VIS; Holland et al., 1980), which has been validated in Portuguese by Santos (2010). The VIS contains 18 true/false items (e.g., “Deciding on a career path has been a long and difficult problem for me”). Santos (2010) found internal consistency estimates of 0.78 and 0.79 for the Portuguese version.

*Career decision* was measured by the *Measurement Scale for Indecisiveness* (MSI; Germeijs and De Boeck, 2002), which measures the ability to make an imminent career decision. The original scale includes 22 items (e.g., “It is easy for me to make decisions”) responded on a 7-point scale ranging from strongly disagree (0) to strongly agree (6). Eleven items are negatively worded (e.g., “It is difficult for me to make a decision”). Rodrigues (2012) validated the MSI to Portuguese and reported an internal consistency estimate (alpha Cronbach) of 0.86.

*Psychological functioning* was measured by the *Beck Depression Inventory-II* (BDI-II; Beck et al., 1996). The BDI-II includes two components: the cognitive-affective (items of 1–13 sections) and the somatic-performance subscales (items of 14–21 sections) (Beck et al., 1996). This inventory was used to assess depressive symptoms with a scale ranging from no depressive symptoms (0) to severe depressive symptoms (63). According to Seggar et al. (2002), clients with scores below 14.3 were considered non-functional and an RCI of 8.46 was used to determine the significant change. A Portuguese validated version of the BDI-II was used (Campos and Gonçalves, 2011). Alpha coefficients in three Portuguese samples are reported as 0.88, 0.90, and 0.91.

*Clients’ distress* was assessed by the *Outcome Questionnaire-45* (OQ-45.2; Lambert et al., 1996a,b). The OQ-45 is composed by 45 items rated on a five-point scale ranging from never (0) to (4) almost always (4), reflecting progress in three dimensions: subjective well-being, interpersonal relationships and social role. Extensive research revealed the good psychometric qualities (reliability and validity) of this measure (Lambert et al., 2004). A Portuguese version of OQ-45 (Machado and Fassnacht, 2014) was used in this study, which reveled adequate psychometric properties for the total score and subscales (α = 0.70–0.92). In the Portuguese version, a score of 0.62 is the cutoff for the clinical population. The clinical cut-off point for this version is 0.62 and the Reliable Change Index (RCI; Jacobson and Truax, 1991) is 0.15.

The TCCS (Ribeiro et al., 2013) was used to analyze the counselor-client dyad therapeutic interactions, in a moment-by-moment basis.

### Procedures

#### Recruitment and Data Collection

The participants were recruited from a university psychology service and from a private practice clinic. All participants were previously informed about the research goals and the procedures included, and all signed an informed consent. Since this study was carried out during a Ph.D., all procedures respected the Ethical Conduct Code of the academic institution where the Ph.D. was developed. CCC occurred according to the phases described by Savickas (2019a). Every intervention session was videotaped and transcribed by two independent trained female post-graduate psychologists, who were 22 and 23 years old and had one and two years of professional experience, respectively. The pre-test occurred 1–6 days before the intervention process. Post-test assessment occurred immediately or until 1 week after the end of the intervention. Each counselor was responsible for all the procedures with each client, individually.

#### Data Analysis

The clinical significance of the intervention was calculated by analyzing pre-post-test statistical differences for each case, with two indexes: Reliable Change Index (RCI = x2–x1/Sdiff) (Jacobson and Truax, 1991) and Zscore (Z*cs* = X – M^N^/DP^N^, Brown, 2015). We considered RCI values equal to or greater than 1.96 as indicative of a clinically significant change. The Zcs standard scores indicate how a client’s score differs from the norm group mean in standard deviation. For all measures, RCI was calculated from the mean score and standard deviation of the normative samples of the Portuguese validation studies.

The sessions codification occurred according to the sequence of procedures of the TCCS manual (Ribeiro et al., 2016b): consensual definition of both judges about the problematic self-narrative of the clients, and of alternative or innovative narrative, individual codification of each counseling session for both judges, and agreement percentage computation and consensual discussion about disagreements. Eight sessions of four clinical cases were independently and sequentially coded until an 80% agreement percentage was obtained for both therapists’ and clients’ utterances. All the sessions of the six cases were coded and analyzed in this study. Cases Cecília, Olga, Vanda and Tatiana had three sessions and cases Nuno and Diana had four sessions.

In addition to the rigor of both the judges training and the codification process, the results’ description was also conducted to ensure the trustworthiness of the study. The writing of the findings, including participants’ quotes, followed the sequence of CCC phases, in order to address the research question, that is: “From the TCM, how does therapeutic collaboration evolve throughout CCC intervention?”. Moreover, the dialogues’ qualitative illustrations were chosen to evidence the fit between data and the authors’ interpretations on the evolution of therapeutic collaboration.

## Results

### Pre–Post-test Results

Table 4 displays the results of the pre-post-test differences in career and clinical measures in the six clients. All the cases presented registered at least one positive statistically reliable change. The cases of Cecília, Olga, and Vanda (pseudonyms) presented statistically reliable change on all the career measures, with only Cecília presenting also a statistically reliable change in OQ-45. In the case of Nuno (pseudonym), there was a statistically reliable and clinically significant change in OQ-45 only. Diana (pseudonym) registered a statistically- reliable change in career certainty and a negative statistically reliable and clinically significant change in OQ-45. Finally, Tatiana (pseudonym) registered a statistically reliable change in career certainty and in vocational identity as well as in BDI-II, and a statistically reliable change and a clinically significant change in OQ-45.

**TABLE 4:** Career and clinical pre-post-test: differences in RCI and Zcs by case.

Case	VCS		VIS		MIS		BDI-II	OQ-45
				
	RCI	Zcs	RCI	Zcs	RCI	Zcs	RCI	RCI
Cecília	2.22**	1,29**	5.22**	3.39**	8.06**	3.31**	5	25**
Nuno	–0.89	–0.52	0.80	0.52	0.18	0.08	7	17*
Diana	2.66**	1.55**	1.61	0.00	0.73	0.30	-6	-19*
Olga	3.99**	2.33**	0.40	1.04**	3.48**	1.43**	0	-4
Vanda	1.77	1.03**	2.81**	1.82**	2.93**	1.20**	1	14
Tatiana	2.22**	1.29**	3.21**	2.08**	1.65	0.68	17**	18*

*Reliable statistical difference and clinically significant.

**Reliable statistical difference.

### Therapeutic Collaboration Results

**FIGURE 2:**
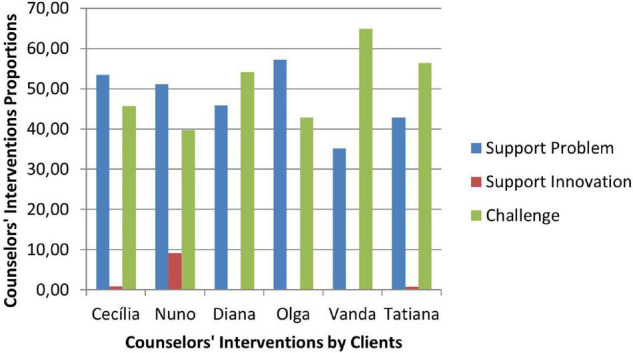
Phase I: counselors’ interventions.

**FIGURE 3:**
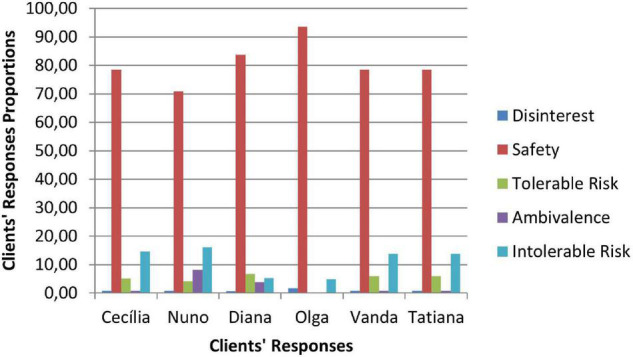
Phase I: clients’ responses.

**FIGURE 4:**
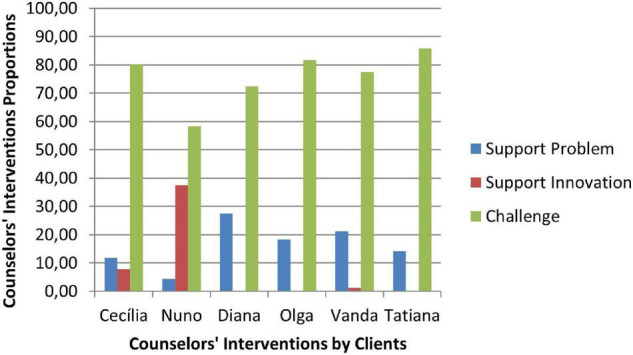
Phase II: counselors’ interventions.

**FIGURE 5:**
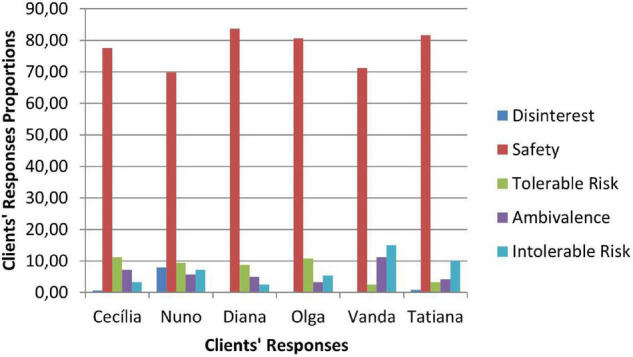
Phase II: clients’ responses.

**FIGURE 6:**
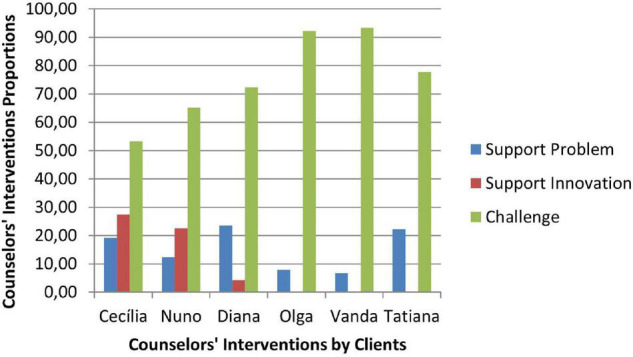
Phase III: counselors’ interventions.

**FIGURE 7:**
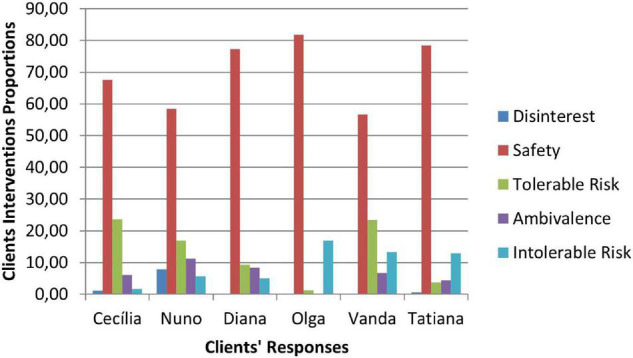
Phase III: clients’ responses.

**FIGURE 8:**
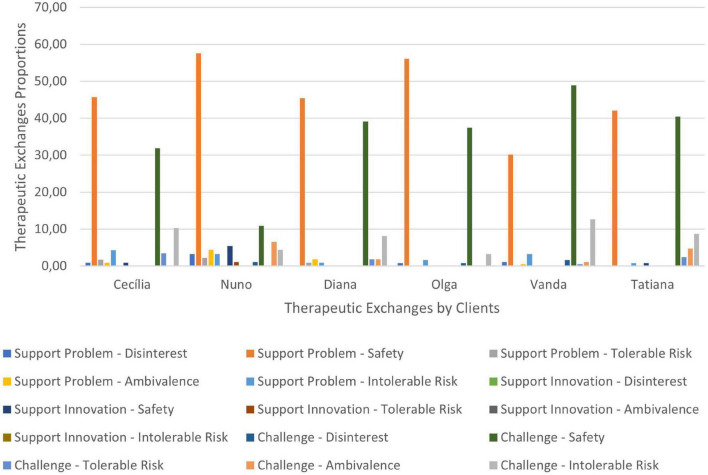
Phase I: therapeutic exchanges.

**FIGURE 9:**
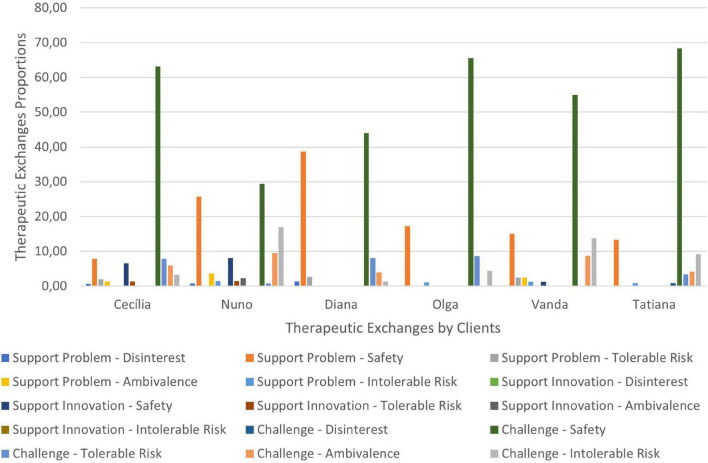
Phase II: therapeutic exchanges.

**FIGURE 10:**
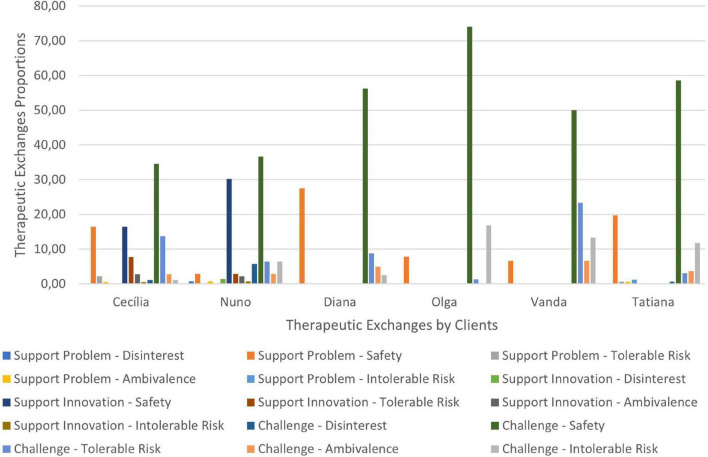
Phase III: therapeutic exchanges.

Therapeutic collaboration results per case for each of the three CCC phases are displayed in Figures 2–10. In Figures 2–7 are presented counselors’ interventions and clients’ responses, separately. In the first phase, the counselor’s Support Problem (SP) and Challenge (C) interventions registered similar percentages. Counselor’s Support Innovation (SI) interventions had almost no expression in this phase (Figure 2). The client’s responses were mostly of Safety (S), with some expression of Intolerable Risk (IR) responses (Figure 3).

The second phase was characterized by a substantial increase of the counselor’s Challenge (C) interventions, a smaller percentage of Support Problem (SP) interventions, and the increase of Support Innovation (SI) interventions (Figure 4). The clients’ responses continued to be mostly of Safety (S), although there was an increase of Tolerable Risk (TR) responses and a decrease in Intolerable Risk (IR) responses (Figure 5).

In the third phase of CCC, the counselor’s interventions of Challenges (C) maintained the higher percentage of occurrence, and the Tolerable Risk (TR) responses occurred more than in the other two phases (Figure 6). The clients responded mostly with Safety (S), Tolerable risk (TR), and Intolerable Risk (IR).

In Figures 8–10 are presented therapeutic exchanges per case for each of the CCC phases. The therapeutic exchanges resulted from the interaction between counselors’ interventions and clients’ responses. In the first phase, interactive episodes of Support Problem-Safety (SP-S) are the most common and followed by Challenge-Safety (C-S) and Challenge-Intolerable Risk (C-IR) episodes. The second phase is characterized by a majority of Challenge-Safety (C-S) episodes and there is an increase of Challenge-Tolerable Risk (C-TR) episodes and a decrease of Support Problem-Safety (SP-S) episodes. There is also a continuity of Challenge-Intolerable Risk (C-IT) episodes. The third phase, interactive episodes continue to be mostly of Challenge-Safety (C-S), followed by Support Problem-Safety (SP-S) and Challenge-Tolerable Risk (C-TR) episodes.

A more detailed explanation of the results regarding the three counseling phases including qualitative illustrations of the cases is presented.

#### Phase I

In phase I, counselors and clients work on career problem formulation and begin narrative elaboration of career constructs (e.g., interests, goals, needs, and values) emerging in elicited career episodes. In this phase, clients need to feel secure to freely explore career problems and evoke career episodes. Counselor’s Support Problem interventions may be adequate at this point, since they help clients reflect on their problems, thoughts and feelings at their current developmental stage (e.g., “What brought you here to this counseling?”; “How can I be useful to you? What kind of help are you looking for?”). Clients in this phase tend to respond almost always with Safety, confirming what the counselor said or providing information. This is congruent with the client’s need for safety, as they articulate, elaborate and express their career narrative (e.g., Vanda – “At this moment, what I really want is guidance on how I should look for a job, because I have a doubt”; Diana – “What I really needed, really and I know it is absolutely unrealistic, was that someone could tell me ‘now you are going to do this, you are going to do this for the rest of your life and that’s it. Settle for it.’ But I was hoping that ah… (2) a counseling really, to try to clarify what I want to do.”). Another task of this phase is the understanding of career constructs, which can develop from the dialogues evoked by CCI questions. The client’s answers to these questions are often of safety, involving the transmission of information, as illustrated in Vanda’s words:

Vanda: “What I am better at, I think is creating empathy, I can create empathy easily. What I am worse at… sometimes I can’t focus or direct the conversation to… to where I should in the time I have, because very often we found interlocutors who speak a lot and ramble a lot and the conversation needs to be directed to where I want, and sometimes it’s difficult for me, I think I have that difficulty.”

At the same time, Challenge interventions also occur in a large proportion. This facilitates the client’s distancing from the problem to construct a new narrative. In this sense, challenging interventions invite the client to deeply explore the causes and consequences of career problems (e.g., “Where does it come from, that need to extend tentacles? (…) Where does this dispersion come from? How does dispersion harm you?”). In this phase, some clients’ responses to challenge are invalidations, mostly because the challenge demands too much, representing an Intolerable Risk for the client’s developmental stage [e.g., Vanda – “I have no idea. I think it is because I like so many things at the same time and I think that is one of my biggest flaws, dispersion”; Nuno – “Well, I don’t know, I don’t know (shakes negatively his head and looks down). It depends. It depends on a lot of things. I don’t know”].

On the other hand, some invalidation responses represent an attitude of disinterest from the client, invalidating the counselor’s intervention, because it doesn’t make sense for the client (e.g., Nuno – “No. I have no idea. I didn’t have that much time to see that.”). Some of the counselor’s challenges in this phase are related to CCI questions. Some of those questions report to childhood and early memories, some of which are difficult to access due to distance in time and associated suffering. The answers to some of these questions are challenging for many clients (Reid et al., 2016), involving Intolerable Risk for the clients’ current level of development. An example is Nuno’s response to the counselors’ question about the first recollection he remembers when he was about 5 or 6 years old:

Nuno: “[Yes.]. When I was 5, 6 years old… (2) I have, I have many flashes of moments and things that… I don’t know, not even my parents remember, not even the majority of people remembers, but… I don’t know, but my memory is also nothing, nothing special, I don’t know why, there are, there are a lot of passages that stay, but I don’t know why.”

Nuno disconnects the topic and doesn’t give an answer because it is very difficult for him to access those memories.

Summarizing, the majority of clients’ responses express safety, especially providing information to the counselors about themselves. After the problems formulation, distance and understanding of career constructs, clients are prepared to reconstruct a new career narrative and a new perspective about themselves.

#### Phase II

During phase II, the major tasks focus on rewriting the career macro-narrative, moving to a new perspective, and developing career plans. The reflection on career constructs emerging from career episodes occurred in different moments and contexts, and fosters the elaboration of the career macro-narrative, structured around a life theme. This continuous process of self-reorganization brings continuity and coherence to the life/career narrative. In this way, narrative identity emerges as the necessary grounds for forming action plans.

Challenge interventions rise abruptly in this phase, unlike Support Problem interventions that decrease and represent a smaller proportion. Counselors and clients now focus on new meanings, linking current and past events to reach a higher level of self-narrative continuity and coherence from which to formulate new career plans. In Vanda’s case, the counselor challenged her to take a new perspective on her problem, suggesting an explanation for her search for balance:

Counselor: “But I have the feeling that you live a little bit between that, your freedom, your autonomy and your need to be with others, and the balance goes a little bit around, is it?”

Other examples are from Cecília’s counselor that was trying to reconstruct a new meaning about her narrative, focused on the insafety Cecília said she felt about the steps to take in the future and on what makes sense to resolve the problem she’s facing:

Counselor: “Hum (…), in fact, it is what stands out more here, the fear, ah, the insafety also in relation to the future, not feeling good, the feeling that, that you fell into a system that you really didn’t like, isn’t it?”
Counselor: “This is the most important above all, isn’t it? That persistence in what you believe, in what you think, eh, that makes sense!”

Clients’ responses continue to be more proportionally focused on safety, emphasizing the clients’ need to work collaboratively in a safe environment while reconstructing a new career narrative and making new career plans [e.g., Cecília: “I already reached that point! (…) It was too fast, I think, but I already reached that point! (laughs)”].

Nevertheless, in phase II there is an increase of Tolerable Risk responses emerging from both the new perspective on career issues and the rewriting of career narrative. Clients make sense of their previous career choices, allowing the development of a new vocational identity and a movement toward change.

For example, the counselor challenges Cecilia to take a new perspective on the problem formulated during the first phase, which allowed her to elaborate about what might be really important:

Counselor: “It can also be this question that, eh, you don’t remember because the important wasn’t what was inside…”
Cecilia: Maybe the process is still more important, isn’t it?

In another example, Tatiana was challenged to reflect on her life motto:

Counselor: “You believe that things will end up solved”
Tatiana: “Yes, yes. I believe that whatever we do good, we’ll receive good, because I think that there are no poor lives, there is no one who has always had a bad life, there must have been at least one good moment and that’s the one to enjoy.”

Making career plans involves a focus on the future and solving career issues through the construction of a new sense of purpose in life/career. The ability to clarify and specify career plans represents an advance in the developmental zone where the risk is tolerable. For example Nuno, who was able to define his preferences for the field of study for a master’s degree:

Nuno: “Yes, exactly! And management and also… accessory and administration.”

Also in this phase, Intolerable Risk responses increase a little to a level similar to those of Tolerable Risk. This might happen since the challenge of rewriting a new self-narrative requires time to advance to a new level of potential development. This was difficult for Tatiana, because she wanted to assume a choice to take a degree in Law, but at the same time, she was struggling to consider other areas of interest, congruent with her family’s expectations.

During phase II, actions supporting Innovation interventions emerge. This type of counseling interventions is justified by the movement, from supporting the problem to challenge being mediated by a space where counselors support and reflect about clients’ emerging self-narrative. In the following vignette, the process is illustrated in dialogue where the counselor supports Nuno’s clarification of his career choice and, next, tries to get more information about that specification:

Counselor (Nuno): “So, Management in the first place, that’s it?” (…) And do you have any idea of what you would need to do? What would be necessary for you to apply?

Conceptually, the movement that starts in the clients’ current development toward their potential development comes from a reflection and understanding of the problem to a reflection about innovations clients bring to counseling and to a challenge, to reflect and understand the career narrative in a new and more adaptive way. However, this movement is not linear. It goes back or forth throughout the whole intervention, and the passage through Support Innovation can be very subtle. This might happen because of the short length of the CCC - three or four sessions in the cases studied – and the consequent counselors need to continue to challenge clients, in order to reach the goals the dyad compromised with in the beginning of the intervention process.

#### Phase III

In the third phase, the major tasks involve reviewing career plans, facilitating its implementation, and the conceptualization of clients’ change. Challenge interventions continue to be the most expressive in this phase, accompanied by a slight rise of Tolerable Risk responses, suggesting the movement toward change. In the next examples, the counselors challenge clients to revise the entry question of how CCC could be useful to the client and to reflect about the conclusion they reached together:

Counselor: “Ok, ahm. So, how do you feel now about (…) the question that brought you initially that was a little bit “having to turn to practice, having to turn to research”?
Cecília: “Well… even yesterday I was thinking about this and (…) because I’m calmer now (…) I realized better that I don’t have to choose one thing or another. I am now doing research and I am enjoying it a lot. Although my goal now is to do investigation, it doesn’t mean it can’t be changed in the future, isn’t it? […] I think I don’t have to have that conflict.”
Counselor (Vanda): “(…) But I was talking about the help, and the help was essentially discovering the social area and somehow spread it a little bit more.”
Vanda: “Yes, because I was very attached to the artistic area and to human resources because it is what I know. The social part was where you opened my range the most.”

Clients are challenged to conceptualize about their transformation and to consider what is different about themselves, leading clients to revise the process and its benefits, like Diana did:

Diana: “I think I really needed this help […] I think it was very therapeutic […] You told me to do things I never thought I had to do, but I had to do them and to think about them and I think it was really good for me.”

Generally, all clients and counselors worked collaboratively, and the sequence of change proposed is based on six cases that presented a reliable change in at least one of the outcome measures. The challenging nature of CCC seems to be an important factor for client’s change, because it fosters innovation. The counselor facilitates change and challenges the client’s potential development. It should be noted that the way clients respond can explain the differences in outcomes. Nuno presented more invalidation responses of Disinterest than any other client, which can help to explain the absence of change in every career outcome measure.

## Discussion

### Therapeutic Collaboration

Therapeutic Collaboration findings evidenced the existence of a pattern of evolving therapeutic collaboration for CCC good outcome cases. The six cases studied were considered successful cases because they presented at least one statistically reliable change in one of the five outcome measures. This pattern that starts with supporting problem interventions in the initial phase and evolves to more challenge interventions in subsequent phases is in line with the pattern found in psychotherapy (e.g., Pinto et al., 2018; Rodrigues, 2018). Counseling in general seems to be characterized by a more supportive phase in the beginning of the intervention to provide a safe environment where clients can work on their problematic self-narratives and evolves progressively to more challenging phases where counselors and clients work collaboratively in clients’ potential developmental level to foster change.

Compared with psychotherapy unsuccessful cases where it is observed that therapeutic work is more focused on clients’ actual developmental level, therapeutic work toward clients’ potential level through challenge interventions and respective clients’ validations is related to successful cases (Rodrigues, 2018). In this sense, the idea that challenge interventions are those that help clients move toward change, is reinforced. Besides, even when counselors and clients work at the same level in co-construction, counselors are considered change enablers (Ribeiro et al., 2013), which seems to happen particularly in CCC.

Although the initial phase is the one where Support Problem interventions mostly occur, therapeutic collaboration results also indicate that challenge interventions are very expressive since the first sessions. These results could indicate that CCC is a very challenging intervention throughout the entire process highlighting the importance of counselors’ factors in the construction of the helping relationship and, in consequence, for change (Heppner et al., 1998).

**FIGURE 11:**
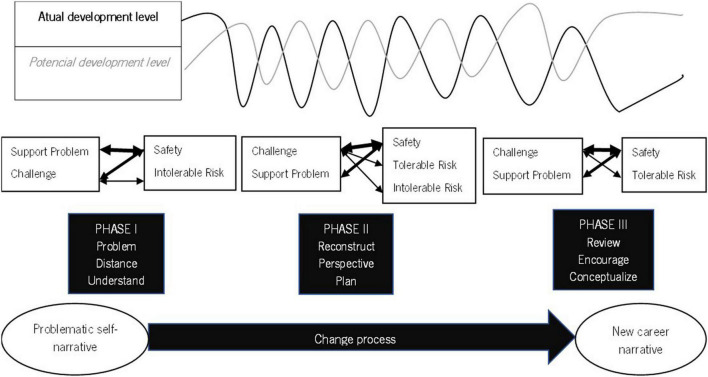
Therapeutic collaboration and tasks in the Construction of Career Change.

Responding to our research question, findings evidenced the existence of a pattern of evolving therapeutic collaboration for CCC. Based on this pattern, we propose a model in which the evolution of therapeutic collaboration during CCC is represented in parallel with the counseling tasks of each CCC phase, as depicted in Figure 11.

The clients’ process of change evolves from the understanding of the problematic self-narrative to the elaboration of a new career narrative. From the Therapeutic Collaboration perspective this narrative transformation evolves throughout the intervention process when clients and counselors work collaboratively in clients’ actual and potential development level.

In phase I counseling tasks foster the presence of challenges in the early moments of the intervention. Clients are asked to respond to the CCI (Savickas, 2019a) questions. Some of these questions report to childhood and to memories not always easy to access, not only because of the time that separates them from the actual moment, but also for its content that could involve suffering episodes and represent, for many people, a challenge. The questions can be themselves challenging considering they are far away from the usual reflection people make about themselves in different life episodes. On the other hand, those questions demand from the clients the access to contents already forgotten or that clients want to avoid. In fact, research on therapeutic collaboration in narrative therapies indicates that, in these interventions, counselors tend to favor challenging interventions (Ribeiro et al., 2011). Clients and counselors work on career problems, distance from them and understand them from a different point of view. Counselors support problem interventions are important to understand clients’ point of view and challenges help clients think differently about themselves. Clients need to feel safe to self-disclosure and some challenges can be too demanding at this phase of the intervention, responding out of their TZPD.

In all cases studied, challenge interventions tend to rise abruptly in the second phase of the intervention process. This is the phase in which counselors and clients reconstruct the new career narrative. This process involves narrative elaboration about the career theme and counselors help clients to give a sense of continuity and coherence to the big narrative of their lives. This process fosters the raising of a new meaning for the career narrative and from that meaning clients can elaborate a new way of looking for the problem and plan a solution. All this process is a challenging exercise for clients. New meanings demand reflection from counselors and clients, what constitutes a challenge to the normal way of feeling, thinking, and acting (Savickas, 2011; Cardoso et al., 2019). Counselors challenge clients to see their narrative in a different perspective and to give a new meaning to their life stories. Clients move forward responding in their potential development level (tolerable risk responses) but need to feel safety to engaged in that movement.

On phase III challenge interventions are the most common once counselors and clients continue to work about the new life narrative. At this phase, the goal is to organize and plan the next career steps (Savickas, 2011; Cardoso et al., 2019). Challenge interventions are important to encourage the implementation of the new plans. Clients and counselors review the new career plans, counselors encourage clients to put them into action and clients reflect upon their own process of change. In this revision and encouragement process, counselors continue to challenge clients and clients continue to need to feel safety while working on their potential development level.

Support innovation interventions are emphasized at this phase because supporting clients’ innovations to the problematic self-narrative means exploring the new emerging narrative (Cardoso et al., 2019) to expand it. When clients innovate, they are at the same level or ahead of their counselors, demonstrating an advance from their starting point. Counselors tend to support clients when they understand that need. Nevertheless, in this research, these supports happen in a lower proportion. This can be explained by the counselors’ need to challenge clients, considering the short length of the intervention and the need to pursue the counseling goals in that period.

Regarding clients’ responses, they follow a similar pattern in all cases. The major proportion of responses are validations by safety throughout all the intervention, a pattern also found in other studies that use TCCS (Ribeiro et al., 2014, 2016a, 2018; Taveira et al., 2017; Pinto et al., 2018).

Clients need to feel safe, understood and validated to reflect deeply about their career narratives (Savickas, 2016; Whiston et al., 2017).

Other validation responses are tolerable risk responses that emerge mostly at the second and third phases of the counseling process. This is an expectable result from TCM (Ribeiro et al., 2013) and from CCC (Savickas, 2011). According to TCM (Ribeiro et al., 2013) counseling is expected to help clients move from their actual to their potential development. This movement starts with more safety responses at the beginning of the intervention, rising gradually tolerable risk responses throughout the intervention process. From CCC (Savickas, 2011) conceptual point of view clients begin working on their problematic self-narrative advancing to the reconstruction of a new narrative that guides the following career plans. The new meaning of life stories, the new way of thinking about the self and the career and the ability to make decisions and plans based on that new narrative are challenges that allow and foster the emergence of tolerable risk responses. Nevertheless, the emergence of more tolerable risk responses as could be expected does not happen due to the short length of the intervention, once clients can differ in the time they need to accommodate the new career narrative and longer interventions could be more beneficial (Cardoso et al., 2016).

Invalidation responses occur mostly in the first and third phases. In phase I clients and counselors are at the beginning of the intervention process, developing a therapeutic relationship. Clients can be unavailable at this phase to respond positively to counselors’ challenges, invalidating their interventions by intolerable risk. On the other hand, at this phase counselors want to understand clients’ concerns, needs and goals, as well as their career stories, supporting and challenging clients in a way not always in line with clients’ thoughts, what could result in responses of Disinterest. This is particularly present in Nuno’s case, the client that presented more Disinterest responses. Nuno revealed to be particularly skeptical about the intervention process what led to this invalidation responses, resulting of more non-collaborative work.

Ambivalence responses occur mostly at the second and third phases. At these phases counselors challenge clients to adopt a new perspective on their career narratives creating an unbalance so clients move on to the reconstruction of the new career narrative. These challenges can create in client’s ambivalence between going further into a new meaning of their life stories and returning to their usual way of thinking, an automatic process that helps to preserve personal consistency (Gonçalves et al., 2011).

### Limitations and Future Research

The proposed sequence of client’s changes results from the study of six cases with different outcomes. One limitation that could lead to future research is the absence of cases that presented no differences in any of the outcome measures studied and dropout cases. It would be useful to understand the therapeutic collaboration pattern in these cases and to compare it with the proposed pattern presented. Another limitation is the absence of control of the counselors’ and client’s characteristics. Some clients seem to be more responsive to challenge (e.g., Vanda) and others were more reluctant to intervention (e.g., Nuno), invalidating more often the counselor. On the other hand, some counselors used more challenge interventions (e.g., Vanda and Tatiana’s counselors) than others (e.g., Diana, Nuno and Olga’s counselor). These differences may be due to the counselor’s age, professional experience and personal style.

### Implications for Practice

The sequence of the client’s change implies some considerations for practice. Due to the importance of the relationship in the therapeutic work, counselors may benefit from self-awareness about the client’s responses in every moment of the intervention process. The TCCS could be an important tool to make counselors more aware of the intervention dynamic in a moment-by-moment basis. If counselors are able to recognize the clients’ invalidations, they could adjust their interventions of Support or Challenge and obtain better outcomes.

In addition, the importance of basic skills of attendance is reinforced. The therapeutic relationship can help clients move toward their potential development fostering change. This relationship depends on the dominance and awareness by counselors of attendance skills like empathy, active listening and responsiveness.

## Data Availability Statement

The raw data supporting the conclusions of this article will be made available by the authors, without undue reservation.

## Ethics Statement

The studies involving human participants were reviewed and approved by the Ethical Conduct Code of the University of Minho. The patients/participants provided their written informed consent to participate in this study. Written informed consent was obtained from the individual(s) for the publication of any potentially identifiable images or data included in this article.

## Author Contributions

FS, MT, and PC contributed to conception and design of the study. ER trained the judges in TCCS and contributed to results analysis and to the revision of the manuscript. FS treated the outcome and process results and wrote the first draft of the manuscript. MT and PC contributed to conceptualization, revision and writing of posterior versions of the manuscript. MS revised the manuscript and gave important inputs to its final version. All the authors contributed to manuscript revision, read, and approved the submitted version.

## Conflict of Interest

The authors declare that the research was conducted in the absence of any commercial or financial relationships that could be construed as a potential conflict of interest.

## Publisher’s Note

All claims expressed in this article are solely those of the authors and do not necessarily represent those of their affiliated organizations, or those of the publisher, the editors and the reviewers. Any product that may be evaluated in this article, or claim that may be made by its manufacturer, is not guaranteed or endorsed by the publisher.
